# Efficacy of Afatinib in the Treatment of Patients with Non-Small Cell Lung Cancer and Head and Neck Squamous Cell Carcinoma: A Systematic Review and Meta-Analysis

**DOI:** 10.3390/cancers13040688

**Published:** 2021-02-08

**Authors:** Nian N. N. Maarof, Abdulsamad Alsalahi, Emilia Abdulmalek, Sharida Fakurazi, Bimo Ario Tejo, Mohd Basyaruddin Abdul Rahman

**Affiliations:** 1 Integrated Chemical BioPhysics Research, Department of Chemistry, Faculty of Science, Universiti Putra Malaysia, Serdang 43400, Selangor, Malaysia; gs53758@student.upm.edu.my (N.N.N.M.); emilia@upm.edu.my (E.A.); bimo.tejo@upm.edu.my (B.A.T.); 2 Department of Chemistry, College of Education, University of Sulaimani, Sulaimani 46001, Iraq; 3 Department of Pharmacology, Faculty of Pharmacy, Sana’a University, Mazbah District, Sana’a Secretariat 1247, Yemen; ahmedsamad28@yahoo.com; 4 Laboratory of Vaccines and Immunotherapeutics, Institute of Bioscience, Universiti Putra Malaysia UPM, Serdang 43400, Selangor, Malaysia; sharida@upm.edu.my; 5 Department of Human Anatomy, Faculty of Medicine and Health Sciences, Universiti Putra Malaysia UPM, Serdang 43400, Selangor, Malaysia; 6 UPM-MAKNA Cancer Laboratory, Institute of Bioscience, Universiti Putra Malaysia, Serdang 43400, Selangor, Malaysia

**Keywords:** afatinib, randomized clinical trials, non-small cell lung cancer, head and neck squamous cell carcinoma

## Abstract

**Simple Summary:**

Evidence from randomized controlled trials about the efficacy of monotherapy of afatinib on survival of patients with advanced non-small cell lung cancer (NSCLC) and recurrent/metastatic head and neck squamous cell carcinoma (R/M HNSCC) has been not yet rigorously reviewed, which needs to be systemically reviewed and meta-analyzed in terms of overall survival and progression-free survival endpoints. The evidence from randomized controlled trials indicated that first- or second-line afatinib monotherapy has improved the survival of patients with NSCLC. Second-line monotherapy afatinib is well-tolerated and could be a promising monotherapy for recurrent/metastatic HNSCCs; however, further randomized controlled trials should be conducted to collect extra survival data regarding the efficacy of afatinib in R/M HNSCC.

**Abstract:**

Several randomized controlled trials (RCTs) evaluated the afatinib efficacy in patients with advanced non-small cell lung cancer (NSCLC) and recurrent/metastatic head and neck squamous cell carcinoma (R/M HNSCC). This review systemically outlined and meta-analyzed the afatinib efficacy in NSCLC and R/M HNSCC in terms of overall survival (OS) and progression-free survival (PFS) endpoints. Records were retrieved from PubMed, Web of Science, and ScienceDirect from 2011 to 2020. Eight afatinib RCTs were included and assessed for the risk of bias. In meta-analysis, overall pooled effect size (ES) of OS in afatinib group (AG) significantly improved in all RCTs and NSCLC-RCTs [hazard ratios (HRs): 0.89 (95% CI: 0.81–0.98, *p* = 0.02); I^2^ = 0%, *p* = 0.71/ 0.86 (95% CI: 0.76–0.97; *p* = 0.02); I^2^ = 0%, *p* = 0.50, respectively]. ES of PFS in AG significantly improved in all RCTs, NSCLC-RCTs, and HNSCC-RCTs [HRs: 0.75 (95% CI: 0.68–0.83; *p* < 0.00001); I^2^ = 26%, *p* = 0.24; 0.75 (95% CI: 0.66–0.84; *p* < 0.00001); I^2^ = 47%, *p* = 0.15/0.76 (95% CI: 0.65–88; *p* = 0.0004); I^2^ = 34%, *p* = 0.0004, respectively]. From a clinical viewpoint of severity, interstitial lung disease, dyspnea, pneumonia, acute renal failure, and renal injury were rarely incident adverse events in the afatinib group. In conclusion, first- and second-line afatinib monotherapy improved the survival of patients with NSCLC, while second-line afatinib monotherapy could be promising for R/M HNSCC. The prospective protocol is in PROSPERO (ID = CRD42020204547).

## 1. Introduction

Non-small cell lung cancer (NSCLC) is a primary histological type lung cancer [1,2,3], which is responsible for 80% to 85% of lung cancer cases [4,5]. On the other hand, the squamous cell carcinoma of head and neck (HNSCC) constitutes the seventh most common global malignancy [6]. However, patients with HNSCC have still been at risk to develop metastases or a second lung squamous cell carcinoma [7] from head and neck cancers, and the metastasis incidence of pulmonary cancer from the head and neck cancer was reported to range from 6–9.1% [8].

Activation of *ErbB* receptors family (e.g., epidermal growth factor receptors; EFGR) initiates a pathway of downstream signaling in cell proliferation, which has a role in glioma progression and germline polymorphism of EGFR [9,10]. Moreover, exons-mutations (exon 19 and 21) for the intracellular EGFR kinase domain render EFGR sensitive to the known EGFR tyrosine kinase inhibitors (TKI) [11]. Interestingly, afatinib is a second-generation TKI that is highly selective on inhibiting *ErbB* family through irreversible binding to EGFR making afatinib more potent and longer acting than reversible first-generation EGFR TKI [12,13].

The chemotherapeutic use of afatinib as a first-line treatment for advance or metastatic EGFR-positive-mutation (EFGR*m*^+^) NSCLC has been approved [14,15,16], while the efficacy of afatinib in treating recurrent/metastatic HNSCC has not been yet approved since activating EGFR*m*^+^ in HNSCC has not been found, despite its overexpression in 90% of HNSCCs, and a minority of patients with HNSCC and NSCLC respond to EGFR-directed inhibition [17,18,19,20]. Nonetheless, EFGR was found to have a role in the treatment of progressed HNSCC, and afatinib has been reported to exert efficacy in R/M HNSCC after platinum-based therapy failure [21], justifying the need for evaluation of the efficacy of afatinib on the survival of patients with either advance NSCLC or R/M HNSCC.

The efficacy of afatinib in treating NSCLC and metastatic/recurrent HNSCC (M/R HNSCC) was reported in several randomized controlled trials (RCTs) in terms of overall survival (OS) and progression-free survival (PFS) [21,22,23,24,25,26,27,28,29,30,31,32]. Interestingly, there is a meta-analysis for the afatinib impact on survival in advanced NSCLC (6 RCTs) and M/R HNSCC (1 RCT) based on the published trials before August 2018 [33]. However, new evidence from more recent RCTs about afatinib impact in NSCLC and M/R HNSCC proposes a need for compiling the updated and previous evidence for afatinib impact on the survival of patients with NSCLC and M/R HNSCC.

Reviewing survival analysis in afatinib-treated patients with NSCLC and M/R HNSCC indicated invariable effects size of afatinib among the published RCTs. Therefore, a hypothesis was assumed that the efficacy of afatinib on survivals of NSCLC and/or M/R HNSCC-patients could be invariable from one RCT to another versus the efficacy of comparable chemotherapies. To examine this hypothesis, the hazard ratios (HRs) with 95% confidence intervals of OS and PFS were obtained from all relevant RCTs as primary endpoints to estimate afatinib efficacy on NSCLC and M/R HNSCC-patients’ survival. Accordingly, the current systematic review and meta-analysis were conducted to identify whether afatinib can modulate OS and PFS in patients with NSCLCs and HNSCCs as compared to standard chemotherapies or placebos in the conducted RCTs

## 2. Materials and Methods

The prospective protocol of this review has been registered in PROSPERO (The International Prospective Register of Systematic Review) [ID = CRD42020204547]. The prospective protocol is accessible online (https://www.crd.york.ac.uk/prospero/display_record.php?RecordID=204547]. These systematic review and meta-analysis adhered strictly to the guidelines of PRISMA-P (the Preferred Reporting Items for Systematic Reviews and Meta-Analyses) [34].

### 2.1. Search Strategy

The search syntax of keywords was: “Afatinib” AND “Non-Small Cell Lung Cancer”; “Afatinib” AND “Squamous Cell Carcinoma of Head and Neck”; “Afatinib” AND “Randomized Controlled Trial”; “Afatinib”. Pertinent records were retrieved from PubMed, Web of Science, and ScienceDirect databases, while the timeframe applied was from 2011 up to August 2020 in all databases. No filters were applied regarding languages or countries to avoid language bias. In the PubMed database, the retrieved records were filtered by Clinical Trial, Phase 1 Clinical Trial, Phase II Clinical Trial, Phase III Clinical Trial, Phase IV Clinical Trial, and RCT, while reviews and books were excluded. In the Web of Science, the selected records were refined by selecting journal articles. In ScienceDirect, the retrieved records were filtered by research articles only. To identify extra records, the search was extended to checklists of references in the published studies, online libraries (Springer, Wiley, Elsevier, MDPI, and J-Stage), National Cancer Institute, U.S. National Library of Medicine (ClinicalTrials.gov), National Technical Information Service (NTIS), International Clinical Trials Registry Platform (ICTRP), Cochrane, and TRIP Medical Database. For grey literature and to avoid publication bias, several open-access databases including MedNar, OpenGrey, and Google Scholar were searched to retrieve unpublished relevant RCTs. For inaccessible articles, the university library or authors were contacted. The total results from each online database and extra sources were recorded in a flow chart diagram of PRISMA. Finally, the number of results from each database was recorded. The retrieval of records was independently performed by two researchers (N.M. and A.A.). Discrepancies were discussed with a third investigator (M.B.A.).

### 2.2. Selection

After removing duplicates, the records that remained were screened by title and abstract to select relevant clinical trials, while books, reviews, meta-analysis, protocols, guidelines, observational studies, and preclinical studies were excluded. Then the remaining records were screened by full text to exclude afatinib-unrelated clinical trials. Finally, the results of the records selection were recorded in the flow chart of PRISMA.

The eligibility criteria (inclusion and exclusion criteria) were specifically tailored in response to pre-specified research question according to PICOS to obtain consistent, relevant evidence (Table 1). The application of eligibility criteria was performed independently by two researchers (N.M. and A.A.). When a consensus was not reached, a third researcher (M.B.A.) was consulted to resolve discrepancies. The number of eligible and excluded records (with reasons) was recorded in the flow chart of PRISMA.

### 2.3. Quality Assessment

#### 2.3.1. Assessment of the Risk of Bias in the Included RCTs

The assessment of the risk of bias was performed at the level of studies. The overall risk of bias across RCTs and each RCT was performed according to the RoB tool of Cochrane utilizing the RevMan software program (Version 5.4; Cochrane Collaboration, Oxford, UK). Two independent investigators (N.M. and A.A.) were required to appraise biases in the included RCTs. Any discrepancies were discussed with a third investigator (M.B.A.) according to the following:

- Selection bias was assessed in terms of “random generation sequences” and “allocation concealment”. If a random generation sequence method (e.g., computer-generated random numbers) was explicitly reported, the result of appraisal was “low risk”. Otherwise, the appraisal was “unclear risk” if the randomization was undertaken without reporting the random generation sequence method or “high risk” if randomization was not undertaken at all. Regarding “allocation concealment”, if it was directly or indirectly reported that the allocation of participants was adequately addressed from time of randomization to the cutoff date of the trial, the appraisal was “low risk”. If it was directly reported that the allocation was concealed (e.g., open-label or single-blind), the appraisal was “high risk” [unless there was a rationale justification]. If not reported directly or indirectly, the appraisal was “unclear risk”.
- Performance bias was assessed in terms of “blinding of participants and personnel”. Therefore, If the masking was double-blind, the appraisal was “low risk”. Alternatively, if it was explicitly reported that the assignment of the trail was an open-label or single-blind, the appraisal was “high risk” [unless there was a rationale justification].
- Detection bias was assessed in terms of “blinding of outcome assessment”. If the outcome assessors and data analysis were reported that performed by an independent review committee, the appraisal was “low risk”. Otherwise, the appraisal was “high risk”.
- Attrition bias was assessed in terms of “incomplete outcome data”. Therefore, if the statistical analysis addressed the dropout, withdrawal, missing data, and discontinuation, the appraisal was “low risk”. Otherwise, the appraisal was “high risk”.
- Reporting bias was assessed in terms of “selective reporting”. Thus, if the primary and secondary outcomes were reported in accord with the objectives of the trial in the published manuscript and prospective protocol of the trial, the appraisal was “low risk”. Otherwise, the appraisal was “high risk” [unless rationally justified].
- Other sources of bias were assessed in terms of “adherence to the prospective protocol”. Therefore, if it was reported that the prospective protocol was not violated during the trial or after interim analysis, the appraisal was “low risk”. Alternatively, if there was direct evidence indicated switching the primary and secondary outcomes, the appraisal was “high risk” [unless rationally justified].

The criteria judged in each RCT were defined by the number of inadequate criteria so that the risk of bias was high when more than 3 domains were high risk indicating that the criteria were adequate to exclude the RCT. Accordingly, the overall reviewer’s judgment to exclude the trail based on the presence of 4 or more high risks of bias [35]. The data that were extracted from the low-biased included RCTs were used in the synthesis of qualitative and quantitative literature reviews.

#### 2.3.2. Quality of the Pooled Evidence from Meta-Analysis

The quality of pooled evidence of meta-analysis was assessed for their up or downgrade according to the tool of GRADE (Grading of Recommendations, assessment, development, and evaluations). The GRADE tool was applied according to the GRADE handbook using GRADEpro.GDT tool. The overall grade estimate could be low, moderate, or serious after grading the domains of study design, indirectness, risk of bias, imprecision, inconsistency, and publication bias. Outcomes were considered to have a high quality of evidence if the certainty in the pooled evidence from each meta-analyzed subset was high.

The quality of the pooled evidence was downgraded, when the pooled endpoints (outcomes) were not pooled from RCTs. Similarly, when the overall risk of bias in all RCTs was high, the quality of the pooled evidence was downgraded. Additionally, the quality of the pooled evidence from each subset was downgraded if they were inconsistent [Non-overlapped confidence intervals and the heterogeneity percentage was high (I^2^ > 50% at *p* < 0.05)]. Moreover, the quality of pooled evidence was downgraded when the measured endpoints were not related to the patients of interest, all the included interventions in RCTs were indirectly relevant, and the interventions and controls were not head to head compared. Furthermore, the evidence was downgraded with detection of imprecise pooled evidence (confidence intervals of each pooled evidence from each subset around the estimate of the effects were wide). Finally, the quality of pooled evidence from each subset was downgraded if publication bias was detected through visualization of the symmetry of RCTs in Funnel plots. Grading the quality of pooled evidence was performed independently by two researchers (N.M. and A.A.). Any discrepancies were discussed with a third investigator (M.B.A.).

### 2.4. Data Collection and Data Extraction Strategy

The data were collected from the included studies, and a standardized evidence table in Microsoft Excel was designed to extract data from the eligible RCTs and the table was named “study characteristics”. The extracted data were reviewed independently by two researchers (N.M. and A.A.). Discrepancies were discussed with a third investigator (M.B.A.).

- Characteristics of study design: Study ID (first author with the year), registered name, prospective protocol ID, interventional model (all the assignment models.), phase, allocation ratio, assignment model, number of centers and countries where the trial was conducted).
- Population: The total number (sample size of participants; age, gender, and ethnicity of participants.
- Characteristics of the health condition of interest: Type and stage of cancer.
- Intervention characteristics: Line of treatment, dose, frequency of dose, route of administration, and the number of participants.
- Comparator characteristics: Type of control (standard therapy or placebo), dose, frequency of dose, route of administration, and number of participants.
- Characteristics of outcomes.
- Primary clinical endpoints: Measure the efficacy of afatinib in terms of participants survival included overall survival (OS) and progression-free survival (PFS), and the data that were extracted included the difference in the effect size (significant or non-significant), hazard ratio with the corresponding 95% confidence interval and P-value, the median of difference and duration of median follow-up of the outcome in weeks or months in the intervention and control groups. OS with median and median follow-up duration [Defined as time from randomization to death for any reason in months and calculated as HR along with its corresponding 95% confidence intervals and P-value]. PFS with median and median follow-up duration [the time from randomization to progression in months and calculated as HR along with its corresponding 95% confidence interval and *p*-value].
- Secondary clinical endpoint was adverse events, which were recruited for evaluating safety (defined as the percentage of severe grade-3 adverse events (AEs) among participants in the intervention as compared to comparator). The data that were extracted included adverse events and the percentage of incidence.

### 2.5. Data Synthesis

#### 2.5.1. Qualitative Synthetic Literature

In a broad scene to evaluate the trend of impact of afatinib on the survival endpoints, the outcomes of OS, PFS, and adverse events from all included RTCs were summarized and criticized. In addition, outcomes of OS, PFS, and adverse events from either NSCLC or HNCSS RCTs were summarized separately.

#### 2.5.2. Meta-Analysis

The meta-analysis was planned for validating conclusions from efficacy outcomes (OS and PFS) if a minimum of three or more RCTs reported the same outcome, while the adverse events were not planned to be enrolled in the meta-analysis. Data were pooled together according to the following subsets: A subset of OS in all RCTs, a subset of OS in NSCLC-RCTs, a subset of OS in HNSCC-RCTs, a subset of PFS in all RCTs, a subset of PFS in NSCLC-RCTs, and a subset of PFS in HNSCC-RCTs. All meta-analyses have proceeded in the RevMan software program (version 5.4; the Cochrane Collaboration, University of Oxford, UK). Firstly, the HRs of either OS or PFS (dichotomous variables) were standardized as hazard ratio ± standard error by using the calculator of RevMan software program as Log10 [hazard ratio]. The Effect size (ES) index was computed for study and then combined and averaged (expressed as weighted for sample size), and 95% confidence intervals of the weighted average effect (positive or negative) were also reported to indicate the precision of the estimate. The I^2^ measure indicated heterogeneity of the pooled difference effect and further assessed visually via the resulted forest plot. During meta-analysis, publication bias was applied using the Funnel plot. Sensitivity analyses were considered through repeating meta-analysis and using the size effect for each subset and statistical comparisons were made across the subsets. To elucidate the effect size, the fixed-effect model was applied assuming that RCTs measure the same intervention with minimal heterogeneity. The effect size difference was assumed to be valid if heterogeneity (I^2^) was more than 50% and *p*-value > 0.1. Otherwise, the random effect size model was proceeded assuming that the trials measured different interventions based on I^2^. Accordingly, if I^2^ was 30–60%, it indicated a moderate heterogeneity, if I^2^ was 50–90%, it indicated a substantial heterogeneity, while if I^2^ was 75–100%, it indicated a considerable heterogeneity. However, if the τ^2^ (tau squared) under the random effect model equaled to zero, this made sense that it did not matter whether either a random or fixed-effect model was used in this meta-analysis. 

The interpretation of the evidence based on the value of the resulting hazard ratio was carried out according to three criteria [36].

- Equivalent efficacy of afatinib to that of the comparator, if hazard ratio = 1.
- Superior efficacy of afatinib to that of the comparator, if hazard ratio < 1.
- Inferior efficacy of afatinib to that of the comparator, if hazard ratio > 1.

## 3. Results

### 3.1. Identification and Selection of Records

**Figure 1:**
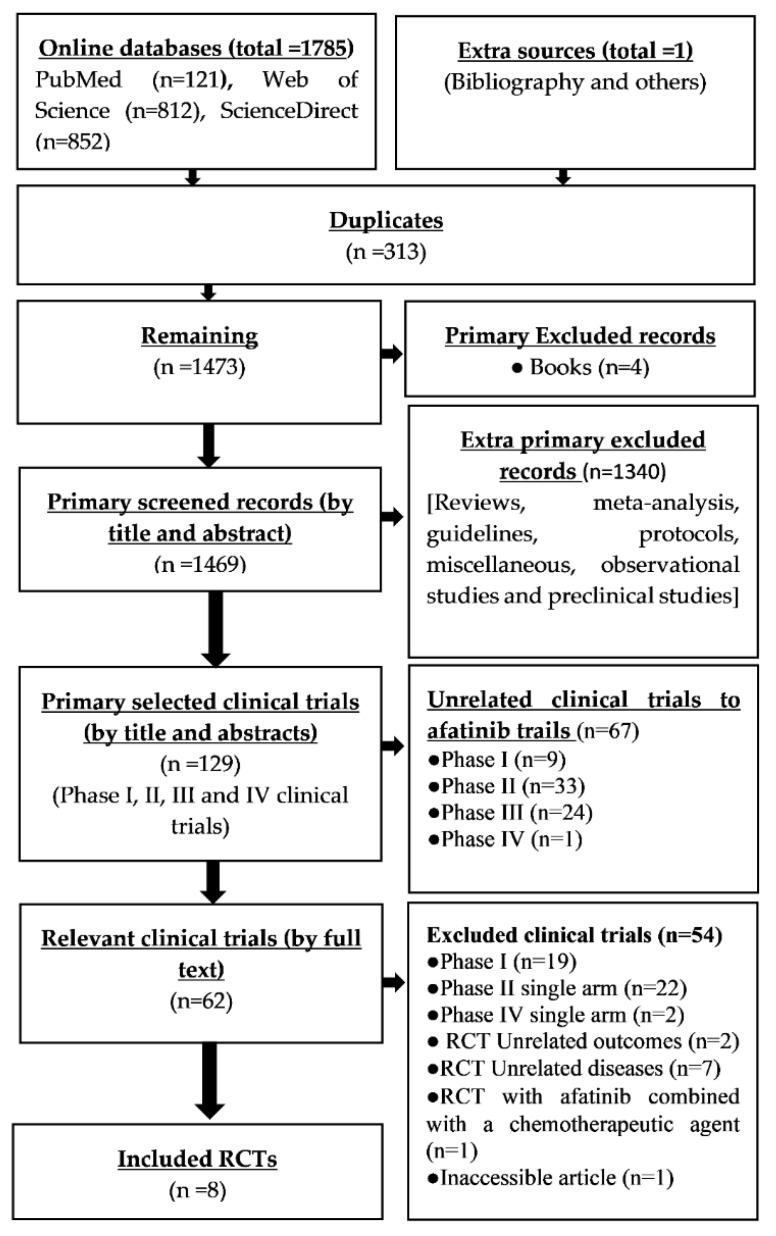
Flow chart of PRISMA.

A total of 1785 records were retrieved from online databases and 1 record from extra sources (Table 2 and Figure 1).

The number of remaining records for primary selection was 1473 after the removal of 313 duplicates. After removing the 4 books, a total of 1469 records remained to be screened by title and abstract. Then a total of 1340 records were excluded (Reviews, meta-analysis, guidelines, protocols, miscellaneous, observational studies, and preclinical studies) to select clinical trials. Accordingly, a total of 129 were primarily identified as clinical trials out of which 67 unrelated clinical trials were excluded. The remaining relevant clinical trials (*n* = 62) subjected to secondary selection through applying the eligibility criteria, out of which 54 clinical trials were excluded for several reasons (Figure 1).

### 3.2. Appriasal of the Risk of Bias within Each RCT and across RCTs

**Figure 2:**
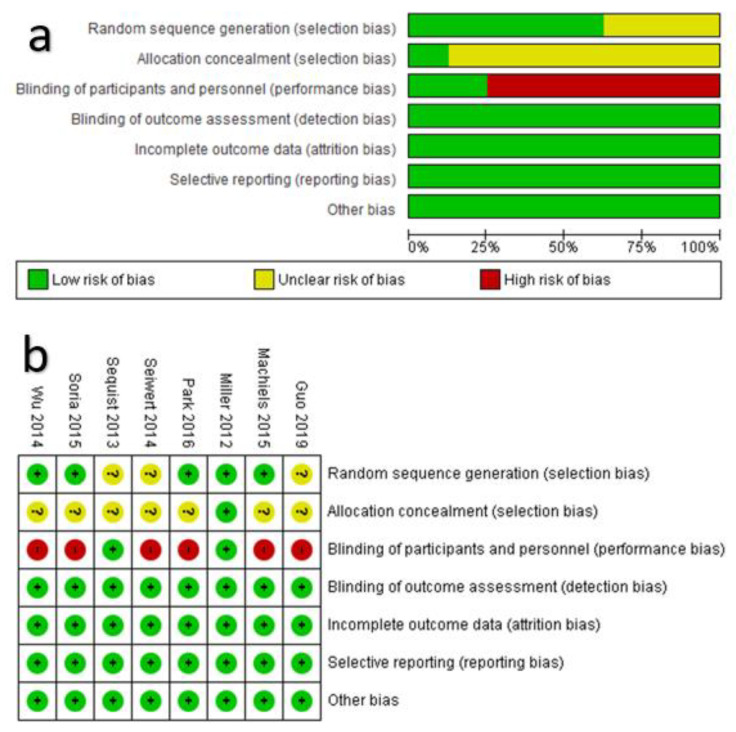
Appraisal of the risk of bias in the included randomized controlled trials (RCTs). (**a**) Total bias risk in all RCTs, (**b**) summary of bias risk in individual RCTs.

The appraisal of the risk of bias was performed at the level of studies. Across the included RCTs (*n* = 8), the blinding of participants and personnel was not adequately addressed, which showed a high risk of bias during the performance. In addition, the allocation concealment was unclear in most of the included studies, while the risks of detection, reporting, attrition, and other sources of bias were low (Figure 2b). Within each study (Figure 2b), six studies out of eight showed a high risk of performance bias due to that the follow-up was open label. Otherwise, the overall reviewer’s judgment showed that risks of bias in selection, detection, attrition, reporting, and other sources of bias in each RCT were low.

### 3.3. Grading the Quality of Pooled Evidence from Meta-Analysis

**Figure 3:**
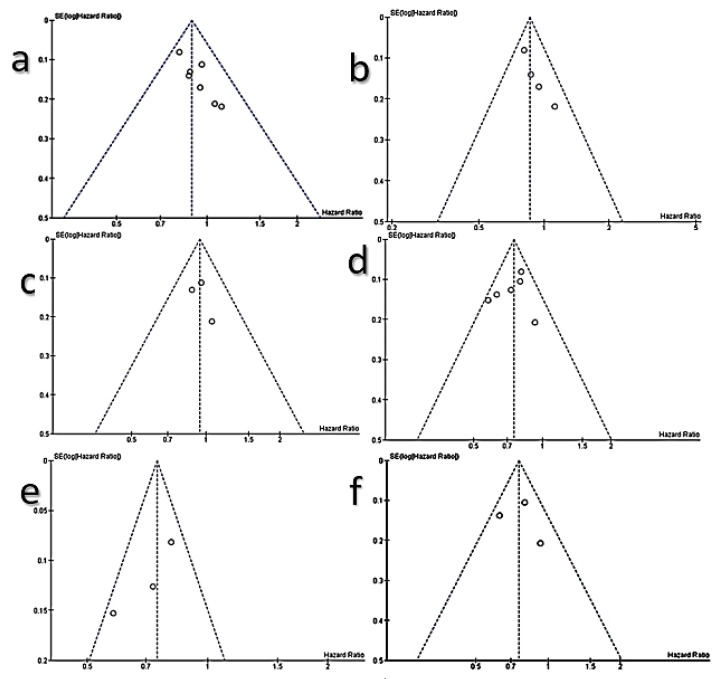
Funnel plots of publication bias; (**a**): overall survival in all RCTs, (**b**): OS in NSCLC RCTs, (**c**): overall survival (OS) in head and neck squamous cell carcinoma (HNSCC) RCTs, (**d**): progression-free survival (PFS) in all RCTs, (**e**): PFS in non-small cell lung cancer (NSCLC) RCTs, (**f**): PFS in HNSCC RCTs.

Certainty in the quality of the pooled evidence of either OS or PFS from meta-analysis was assessed at the level of outcomes in the enrolled RCTs after application of the sensitivity test across RCTs in each subset. The results showed that the certainty in the quality of pooled evidence of either OS or PFS was high, (Table 3). Publication bias was not detected in all subsets, which showed that publication bias did not affect the cumulative evidence (Figure 3).

### 3.4. Study Characteristics

Regarding the included studies in the systematic qualitative literature, eight parallel afatinib-relevant interventional RCTs were included in this systematic review [22,23,24,25,26,30,31,32]. Five RCTs investigated the efficacy and safety of afatinib against placebo or standard chemotherapy in patients with advanced (stage IIIB or IV) NSCLC [22,23,24,25,26], while three RCTs investigated the efficacy and safety of afatinib against standard chemotherapy in patients with M/R HNSCC [30,31,32]. On the other hand, all the included RCTs were multicenter involving hundreds of centers (*n* = 645) in several countries and covered a time frame from 2012 to 2019. Regarding the recruited population, the included RCTs enrolled 3352 patients distributed as 2408 patients with NSCLC and 944 patients with M/R HNSCC, who received an oral single daily dose of either 40 or 50 mg of afatinib (monotherapy) against placebos or standard chemotherapies. Moreover, the enrolled participants were 18 years old and more who belonged to both genders with multiethnicity including Eastern Asians (Chinese, South Korean), South-East Asians, other Asians, White, Black/African American, American Indian/Alaska (natives).

Regarding the included RCTs in the meta-analysis (quantitative literature), seven out of eight RCTs were enrolled into the meta-analysis to evaluate OS in the subset all RCTs (patients with advanced NSCLC and metastatic/recurrent HNSCC) [23,24,25,26,30,31,32] covering 2762 participants (≥18 years, both gender, multiethnicity). On the other hand, six out of eight RCTs were enrolled into the meta-analysis to evaluate PFS in the subset all RCTs (patients with advanced NSCLC and metastatic/recurrent HNSCC) [23,25,26,30,31,32] covering 2398 participants (≥18 years, both genders, multiethnicity). 

In the evaluation of OS in NSCLC-RCTs, 4 RCTs were included (patients with advanced NSCLC) [23,24,25,26] covering 1818 participants (≥18 years, both gender, multiethnicity), while three HNSCC-RCTs [30,31,32] were enrolled to evaluate the OS in patients with M/R HNSCC covering 944 patients ((≥18 years, both gender, multiethnicity).

In the evaluation of PFS in NSCLC-RCTs, three RCTs were included (patients with advanced NSCLC) [23,25,26] covering 1454 participants (≥18 years, both gender, multiethnicity), while 3 HNSCC-RCTs [30,31,32] were enrolled to evaluate the PFS in patients with M/R HNSCC covering 944 patients ((≥18 years, both gender, multiethnicity). (Appendix A).

### 3.5. Qualitative Literature Review

#### 3.5.1. Efficacy of Afatinib

The RCT reported by Miller et al. [22] was the first RCT that assessed the efficacy of afatinib monotherapy (second-line) for progressed advanced NSCLC after the failure of the treatment with gefitinib or erlotinib. The RCT by Miller et al. [22] was phase III, double-blind, parallel, and multicenter (86 centers in 15 countries) RCT, which recruited multiethnic participants (East Asian, other Asians, and others from several Asian, European, and North American countries) with different ages (18 years and older; both genders). In terms of intention-to-treat analysis, the RCT by Miller et al. [22] recruited 585 participants, who were randomly enrolled in the intervention (*n* = 390) and control (*n* = 195) groups, receiving 50 mg afatinib (single oral daily dose) with a supportive best care or a daily oral placebo with a supportive best care. For estimating the impact of afatinib on survival, the RCT by Miller et al. [22] was statistically powered to measure the OS as primary endpoint assuming that OS in the afatinib arm would be shorter than that of the placebo. The results of the RCT by Miller et al. [22] showed that OS median follow-up in the afatinib arm (10.8 months) was not different from that in the placebo (12 months), and the HR was 1.08 (95% CI: 0.86–1.35), which indicated that the Priori hypothesis about OS as a primary endpoint was rejected. In contrast, PFS was recruited as a secondary endpoint with 3.3 months median follow-up in the afatinib arm that was significantly longer as compared to placebo (1.1 months), and the HR was 0.38 (95% CI: 0.31–0.48). The former findings indicated that PFS was more successful than OS in estimating the survival of patients. In terms of PFS, the RCTs by Miller et al. [22] established that afatinib could be beneficial in the second-line chemotherapy for the progressive TKI-resistant NSCLC.

The RCT by Sequist et al. [23] examined a priori hypothesis of the superiority of first-line afatinib chemotherapy in patients with EFGRm+-NSCLC to pemetrexed-based chemotherapy. This RCT was phase III, parallel, multicenter (133 sites in 25 countries), and open-label, which recruited multiethnic (white and Eastern Asian), naïve-treatment participants, with advanced NSCLC at different age (18 years and older; both genders). In terms of intention-to-treat-analysis, the RCT by Sequist et al. [23] randomly allocated 345 participants in intervention (*n* = 229) and control (*n* = 111) who received 40 mg afatinib (single daily oral dose) or cisplatin (intravenous 75 mg/m^2^) plus pemetrexed (intravenous 500 mg/m^2^; treatment cycle of once every 21 days for 6 cycles), respectively. For estimating participants’ survival, the RCT by Sequist et al. [23] used PFS as a primary endpoint to estimate the survival of patients, while OS was the secondary endpoint. This RCT was statistically powered to measure PFS (at the time of primary analysis) assuming an HR of 0.64 with a 7-month median follow-up for chemotherapy and an 11-month median follow-up for afatinib. This RCT was powered statistically to detect OS upon observing 209 deaths events. At the cutoff date, the results of the RCT by Sequist et al. [23] showed that the PFS median follow-up in the afatinib arm was 11.1 months that was significantly longer than the median follow-up in the control (6.9 months), and the HR was 0.58 (95% CI: 0.43–0.78). At the cutoff date, on the other hand, only 98 participants died, which indicated an immature OS (at the time of primary analysis of PFS) due to that the median follow-up of OS in the afatinib (16.6 months) group was not significantly longer as compared to control (14.8 months) [HR: 0.60 (95% CI: 0.73–1.54)]. It seems that the former findings of OS and even PFS in the RCT by Sequist et al. [23] were consistent with the earlier results of OS and PFS in the RCT by Miller et al. [22]. Finally, it could be concluded that PFS was successful (as a primary endpoint) in establishing that the first-line treatment of naïve EFGR*m*^+^ NSCLC patients with afatinib was superior to the platinum-based chemotherapy. However, OS failed to establish that the patients in the afatinib arm lived longer than those who received standard double chemotherapy.

The RCT by Wu et al. [24] gathered further data regarding the efficacy and safety of afatinib in advanced EFGRm+ NSCLC. This RCT was a phase III, parallel, multicenter (36 centers distributed between Thailand, China, and South Korea), open-label RCT. However, the RCT by Wu et al. [24] was different from the RCTs by Miller et al. [22] and Sequist et al. [23] in terms of ethnicity settings through recruiting only Asian participants from the viewpoint that mutant EGFR is more common in Asians than non-Asians [24]. Accordingly, the RCT by Wu et al. [24] specified its aim to make a comparison between the efficacy of afatinib (first-line treatment) in EFGRm+ NSCLC Asians against gemcitabine plus cisplatin chemotherapy that is widely recommended in Asian countries. In terms of intention-to-treat analysis, the RCT by Wu et al. [24] randomly allocated 364 participants in the intervention (*n* = 242) and control (*n* = 122) groups receiving a single oral daily dose of 40 mg of afatinib or gemcitabine (intravenous 1000 mg/m²) with cisplatin (intravenous 75 mg/m^2^) in a three-week schedule, respectively. For estimating the survival of patients, the RCT by Wu et al. [24] was statistically powered to recruit PFS as a primary endpoint assuming that among the 330 enrolled participants, it would be a minimum 217 PFS observed death events with an HR of 0.64 and an 11-months follow-up median with afatinib versus a 7-months with gemcitabine plus cisplatin chemotherapy. On the other hand, OS was applied as a secondary endpoint, which was planned to be adequately matured after observing 237 death events at the time of primary analysis. The results of RCT by Wu et al. [24] showed that the median follow-up of PFS in the afatinib (11 months) group was significantly longer than that in the control (6.5 months) group with an HR of 0.26 [95% CI: 0.19–0.36] indicating that Priori hypothesis was achieved and the efficacy of afatinib (first-line treatment) in EFGR*m*^+^ NSCLC Asians was superior to that of gemcitabine plus cisplatin chemotherapy. Conversely, the median follow-up of OS in the afatinib arm (22.1 months) was similar to that in the control (22.2 months) group [HR of 0.95 (95% CI: 0.68–1.33)].

The RCT by Soria et al. [25] was a phase III, parallel, multicenter (183 sites in 23 countries), and open-label RCT that that recruited Asian patients (Non-eastern Asian and Eastern Asian) with an advanced NSCLC similar to the RCT by Wu et al. [24]. This RCT aimed to compare the efficacy of afatinib (second-line treatment) in NSCLC against erlotinib. In terms of the intention-to-treat analysis, the RCT by Soria et al. [25] randomly allocated 795 multiethnic worldwide participants (18 years and older; both genders) in the intervention (*n* = 398) and control (*n* = 397) groups receiving a single oral daily dose of 40 mg of afatinib or a single daily oral dose of 150 mg of erlotinib, respectively. In estimating the patients’ survival, PFS was used as a primary endpoint, while OS was used as a secondary endpoint. The RCT by Soria et al. [25] was statistically powered to measure PFS assuming that 372 PFS events would be observed with a median follow-up in the afatinib arm (3.2 months) as compared to 2.3 months in the control (erlotinib) [HR: 0.714]. For OS, the RCT was planned to observe 632 death events with an 8.8-months follow-up median in the afatinib arm as compared to 7 months in the control [HR: 0.80]. The RCT by Soria et al. [25] showed that the median follow-up of the PFS in the afatinib arm (2.6 months) was significantly longer as compared to erlotinib (1.9 months) [HR: 0.81 (95% CI: 0.69–0.96)], while the median follow-up of OS in the afatinib arm (7.9 months) was significantly longer as compared to erlotinib (6.8 months) [HR: 0.81 (95% IC: 0.69–0.95)]. Accordingly, both PFS and OS were of close HRs, which means that patients on afatinib lived longer than those on erlotinib. In addition, the Priori hypothesis of this RCT was achieved to establish that the efficacy of afatinib with an advanced NSCLS was superior to that of erlotinib.

The RCT by Park et al. [26] was a phase II, parallel, multicenter (64 sites in 13 countries) and open label, which recruited participants with advanced EFGRm+ NSCLC who were 18 years and older (both genders), multiethnic (Asian, Black/African American, and White). This RCT aimed to make a comparison between the efficacy of afatinib (first-line treatment) and gefitinib in EFGRm+ advanced NSCLC. In the intention-to-treat analysis, this RCT randomly allocated 319 participants into an intervention (*n* = 160) and a control (*n* = 159) receiving a single oral daily dose of 40 mg afatinib or 250 mg gefitinib, respectively. In the RCT by Park et al. [26], both PFS and OS were recruited as primary endpoints, which was statistically powered to observe 250 PFS events [HR: 0.25] as well as 213 OS events with a 32-months median follow-up. The results of RCT by Park et al. [26] showed that the median follow-up of PFS in the afatinib arm (11 months) was significantly longer as compared to control (10.9 months) [HR: 0.73 (95% CI: 0.57–0.95)], while the OS median follow-up in the afatinib arm (27.9 months) was similar to that in the control (25 months) [HR: 0.87 (95% CI: 0.66-1.15)]. In the RCT by Park et al. [26], the OS was not mature to estimate the patients’ survival in the afatinib arm against control, while PFS was successful in establishing that the efficacy of first-line treatment of advanced NSCLS with afatinib was superior to that of gefitinib. 

The RCT by Seiwert et al. [30] was a phase II, parallel, multicenter (43 sites in 4 countries) and open-label RCT recruiting participants with different ages (18 years and older, both genders) and multiethnic (Black, White, American Indian/Alaska, Natives). This RCT aimed to assess whether afatinib would have superior efficacy to cetuximab in treating R/M HNSCC that was progressed after platinum-containing chemotherapy. In terms of intention-to-treat analysis, the RCT by Seiwert et al. [30] randomly allocated 121 participants in an intervention (*n* = 61) and a control (*n* = 60) receiving a single oral daily dose of 50 mg of afatinib or once-weekly 250 mg/m^2^ cetuximab, respectively. To measure the survival of participants, PFS and OS were measured as secondary endpoints; however, this RCT was not statistically powered to measure PFS and OS. The results of the RCT by Seiwert et al. [30] showed that PFS median follow-up in the afatinib arm (13 weeks) was rather shorter than that in the control (15 weeks) group [HR: 0.93 (95% CI: 0.62–1.38)]. Similarly, the OS median follow-up in the afatinib (35.9 weeks) group was rather shorter than that in the control (47.1 weeks) with an HR of 1.06 [95% CI: 0.70–1.62]. Accordingly, both PFS and OS were not conclusive regarding the establishment of the beneficial impact of afatinib on the survival of patients with R/M HNSCC.

The RCT by Machiels et al. [32] was phase III, parallel, multicenter (101 sites in 19 countries), and open label, which recruited participants with different ages (18 years and older; both genders) and multiethnic. This RCT assessed the efficacy and safety of afatinib (second-line treatment) in patients with R/M HNSCC after platinum-based therapy versus methotrexate. In the intention-to-treat-analysis, this RCT randomly allocated 483 participants in an intervention (*n* = 322) and a control (*n* = 161) receiving a single oral daily dose of 40 mg of afatinib or an intravenous once-weekly dose of 40 mg/m^2^ methotrexate, respectively. For survival analysis, PFS was applied as a primary endpoint, while OS was applied as a secondary endpoint for them the RCT was statistically powered to randomize 474 patients. The results demonstrated that the PFS median follow-up in the afatinib (6.7 months) group was significantly longer as compared to control (2.6 months) [HR: 0.80 (95% CI: 0.65–0.98)], while the OS median follow-up in the afatinib (6.8 months) group was similar to that in the control (6 months) group [HR: 0.96 (95% CI: 0.77–1.19)]. Accordingly, PFS was successful to establish that the efficacy of afatinib (second-line treatment) was superior to methotrexate in terms of prolongation of the time to progression of R/M HNSCC.

The RCT by Guo et al. [31] was a phase III, parallel, multicenter (53 sites in 8 countries), and open-label RCT, which recruited participants with different ages (18 years and older; both genders) and multiethnic (Asian and White) to compare the second-line treatment with afatinib against methotrexate in R/M HNSCC. In the intention-to-treat analysis, this RCT randomly allocated 340 participants in an intervention (*n* = 228) and a control (*n* = 112) receiving a single oral daily dose of 40 mg of afatinib or an intravenous once-weekly dose of 40 mg/m^2^ methotrexate, respectively. For survival analysis, PFS was applied as a primary endpoint; while OS was applied as a secondary endpoint. The study was statistically powered to observe 274 PFS progression/death events with a 3-month median follow-up with afatinib and 2.1 months with methotrexate. The results showed that the median follow-up of PFS in the afatinib arm (2.9 months) was significantly longer as compared to control (2.6 months) [HR: 0.63 (95% CI: 0.48–0.82)], while OS was similar to that in the control group [HR: 0.88 (95% CI: 0.68–1.13)]. Accordingly, PFS was successful to establish that the efficacy of afatinib (second-line treatment) was superior to that of methotrexate in terms of prolongation of the time to progression of recurrent metastatic HNSCC.

#### 3.5.2. Adverse Events

The most common grade 3 AEs that have been reported in the included studies were Interstitial lung disease [32], dyspnea [22,32], Pneumonia [26,32], acute renal failure, and renal injury [26,32]. Although the percentages of incidence of all the grade 3 adverse events were very low (15–0.4%), they have been still fatal and need hospitalization.

### 3.6. Meta-Analysis

It should be clear that meta-analyses were performed by proceeding the fixed effect model with the application of the sensitivity test across RCTs of each subset. The extracted HRs of either OS or PFS from RCTs are shown in (Appendix B).

**Figure 4:**
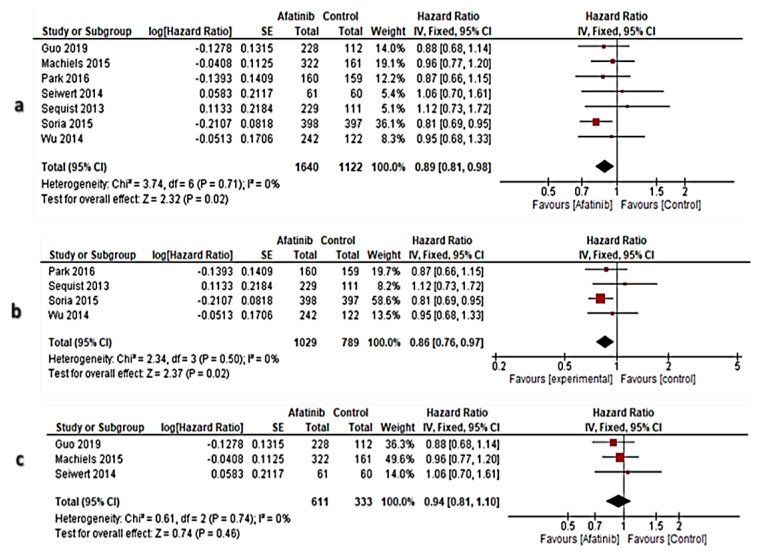
Forest plots of OS; (**a**): Subset of OS in all RCTs, (**b**): The subset of OS in NSCLC, (**c**): subset of OS in HNSCC. Fixed effect model of meta-analysis has proceeded. Red color is the CenterPoint of the confidence interval expressing the weight of the sample size in each RCT, while black-colored diamond denoted the overall effect size.

In all RCTs, OS evidence from RCT by Miller et al. [22] was excluded after application of the sensitivity test across the eight RCTs. Therefore, the meta-analysis enrolled seven RCTs [23,24,25,26,30,31,32]. The results showed that the overall pooled effect size of OS in the afatinib arm was significantly improved versus controls [HR: 0.89 (95% CI: 0.81–0.98) at *p* = 0.02], and the heterogeneity was non-significantly different (I^2^ = 0% at *p* = 0.71) (Figure 4a).

A meta-analysis of the OS evidence from NSCLC-RCTs was intended to enroll five RCTs, however, one RCT was excluded [22] after application of the sensitivity test. Therefore, four RCTs [23,24,25,26] were enrolled in the meta-analysis. Accordingly, the overall pooled effect size of OS in afatinib arm was significantly improved versus controls [HR: 0.86 (95% CI: 0.76–0.97) at *p* = 0.02], and the heterogeneity was non-significantly different (I^2^ = 0% at *p* = 0.50) (Figure 4b).

In the subset of OS in HNSCC, three RCTs [30,31,32] were enrolled, and the results showed that the overall pooled effect size of OS in the afatinib arm was not significantly improved versus controls [HR: 0.94 (95% CI: 0.81–1.10) at *p* = 0.46], and the heterogeneity was non-significantly different (I^2^ = 0% at *p* = 0.74), (Figure 4c).

**Figure 5:**
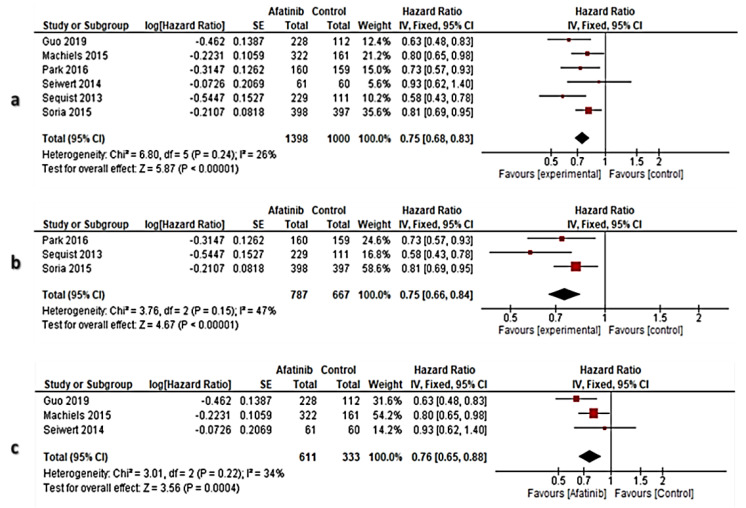
Forest plot of PFS; (**a**): Subset of PFS in all RCTs, (**b**): The subset of PFS in NSCLC, (**c**): The subset of PFS in HNSCC. Fixed effect model of meta-analysis has proceeded. Red color is the CenterPoint of the confidence interval expressing the weight of the sample size in each RCT, while black-colored diamond denoted the overall effect size.

In the subset of PFS in all RCTs, the PFS evidence from two RCTs [22,24] was excluded after application of the sensitivity test across the eight RCTs. Therefore, the meta-analysis enrolled six RCTs [23,25,26,30,31,32]. Hence, the results showed that the overall pooled effect size of PFS in the afatinib arm significantly improved PFS versus controls [HR: 0.75 (95% CI: 0.68–0.83) at *p* < 0.00001], and the heterogeneity was non-significantly different (I^2^ = 26% at *p* = 0.24) (Figure 5a).

A meta-analysis of the PFS evidence from NSCLC-RCTs was intended to enroll five RCTs, however, two RCTs were excluded [22,24] after application of sensitivity test across the enrolled RCTs. Therefore, three RCTs [23,25,26] were enrolled in the meta-analysis. Hence, the results showed that the overall pooled effect size of PFS in the afatinib arm was significantly improved versus controls [HR: 0.75 (95% CI: 0.66–0.84) at *p* < 0.00001], and heterogeneity was non-significantly different (I^2^ = 47% at *p* = 0.15) (Figure 5b).

In the subset of OS in HNSCC, three RCTs [30,31,32] were enrolled. Accordingly, the results showed that the overall pooled effect size of PFS in the afatinib arm was significantly improved versus controls [HR: 0.76 (95% CI: 0.65–88) at *p* = 0. 0.0004], and the heterogeneity was non-significantly different (I^2^ = 34% at *p* = 0.0004), (Figure 5c).

## 4. Discussion

This review systematically summarized the survival evidence of patients with NSCLC and HNSCC, followed by a meta-analysis to validate those findings of the qualitative literature. This review enrolled nine eligible RCTs following rigorous methodologies to draw high-quality conclusion [37].

The qualitative literature indicated that OS was not fully successful to estimate the survival of patients in seven out of the eight included RCTs due to the medians follow-up of OS in the afatinib arm were similar to medians follow-up in the controls [22,23,24,26,30,31,32], while PFS was successful in estimating the survivals of patients in six out of the included RCTs due to the medians of follow-up in the afatinib arm was longer than those in the controls [22,23,24,25,26,31,32]. These findings could indicate that performance of PFS in estimating the survival of patients was more efficient than that of OS, which could be due to the immaturity of OS at the time of analysis [23,24,26], the cancer treatments received by some participants after progression [22,32], or the RCTs were not powered to detect benefits of OS [30,31]. Conversely, the efficient performance of PFS could be due to that PFS had not affected by the mixed chemotherapies [33]. Nonetheless, the findings from the meta-analysis regarding the overall pooled effect size of OS in seven out of eight RCTs indicated that afatinib significantly improved the survival of patients in the afatinib arm [23,24,25,26,30,31,32], a finding that is consistent with that was reported by a pervious meta-analysis [33].

The findings from meta-analyses of the OS in the subset of NSCLC patients in four out of five RCTs [23,24,25,26] indicated that the overall pooled effect size of OS significantly improved the survival of patients in the afatinib arm as compared to that in the control group, while the overall pooled effect size of OS in the meta-analysis of the three HNSCC-RCTs indicated that afatinib could not improve the survival of patients with recurrent metastatic HNSCC. While most of the NSCLC study is performed in the first-line setting, all three HNSCC studies were conducted in the second-line setting. This might explain why OS was not significant among the HNSCC studies.

Regarding PFS, the overall pooled size effect in six out of eight RCTs indicated that afatinib significantly prolonged the survival of patients with NSCLC and HNSCC, which is consistent with that was reported in a previous meta-analysis [33]. Similarly, the overall pooled effect size of PFS from the meta-analysis of PFS in three out of five RCTs of NSCLC and the three HNSCC-RCTs indicated that afatinib improved patients’ survival.

The most common severe grade-3 AE that have been reported among the participants in the afatinib arm in the included RCTs were interstitial lung disease, dyspnea, pneumonia, acute renal failure, and renal injury. Although the percentages of incidence of all the grade-3 adverse events were very low (15–0.4%), clinicians should consider precautions when prescribing afatnib for patients with other respiratory and/or renal co-morbidities because these AE may be fatal and need hospitalization. Due to the incidence of AEs in the included RCTs was not estimated with an estimator (such as odd ratio), we could not proceed meta-analysis for the reported AEs. However, further network meta-analysis of the adverse events for the different TKIs would be more practical to make the choice of TKIs dependent on their adverse event profiles [38]. Nonetheless, there are major challenges for systematic reviews evaluating adverse effects due to high diversity in the number and type of possible AEs, as well as variation in their definition, methods of ascertainment, incidence, and time-course.

This systemic review recruited PFS and OS as primary endpoints to estimate the survival of patients with NSCLC and HNSCC due to that PFS and OS are definitive endpoints in terms of the clinical benefits as well as OS and PFS are objective endpoints of which measurement does not prejudice or favor [39].

As a limitation of the included RCTs in this systemic review and meta-analysis, a total of six out of the eight eligible RCTs were open label, which could indicate that the blinding of participants and investigators was unsatisfactory. However, the bias in all eligible RCTs was low. Noteworthily, the open-label follow-up of the participants was intentionally planned in most RCTs, which could be due to the issue of participants’ safety [39], the need for these RCTs to gather additional information about the long-term effects of afatinib [40], or the nature of the interventions may not permit the application of blinding [41]. Even if the allocation was mostly concealed in the eligible RCTs, the assessment of objective endpoints (OS and PFS) was performed by independent review committees, which could minimize the risk of blinding bias [41]. 

As a limitation of the current systemic review and meta-analysis, efficacy evidence and AEs from the observational studies were not included. However, this systemic review included RCTs since the grade of quality of the pooled evidence from RCTs is high [42]. Hence, the appraisal of the certainty of the quality of the pooled evidence of OS and PFS in this review was high in all subsets, which could power the conclusions of the current systemic review and meta-analysis. Moreover, subgroup analysis was not implemented to elucidate the effect of the difference of ethnicity of participants on the efficacy of afatinib on the survival of patients with either NSCLC or HNSCC because the recruited multiethnicities were not consistent across the included RCTs, which would make the subgroup analysis of OS and PFS by ethnicity inconsistent to draw an informative conclusion. However, further Individual Patients Meta-analysis in each group should be considered in the future.

## 5. Conclusions

First or second-line monotherapy with a single oral daily dose of 40 mg of afatinib is beneficial and improves the survival of patients with advanced NSCLC and recurrent/metastasis HNSCC. In addition, administration of afatinib could be rarely associated with fatal or serious AEs such as interstitial lung disease, which indicates that afatinib should be precautious prescribed. Moreover, afatinib could be a promising effective single chemotherapy for recurrent/metastatic HNSCCs; however, further RCTs should be conducted to collect extra survival data regarding the efficacy of afatinib in recurrent metastatic HNSCC.

## Figures and Tables

**Table 1:** Eligibility criteria for secondary records selection.

Items	Inclusion	Exclusion
Study design	^1^ RCTs (phase II, Phase III, any design, from 2011-2020, any length of follow-up)	Phase I clinical trials and phase II or IV single-arm clinical trials. RCTs with unrelated outcomes RCTs with cancers other than NSCLC or M/R HNSCC.
Health problem	^2^ NSCLC or M/R ^3^ HNSCC (any stage)	Cancers other than NSCLC or M/R HNSCC
Population	Humans patients with NSCLC or M/R HNSCC (any age; gender or ethnicity)	Healthy humans Humans with other types (s) of cancer
Intervention	Afatinib (line of treatment, orally administered, any dose, any dose frequency, any timing)	Afatinib combined with a chemotherapeutic or non-chemotherapeutic agent(s) Surgery or radiotherapy Administered via a route other than oral route
Comparator	Chemotherapy or placebo (Route of administration, any dose, frequency, timing)	Natural supplements, surgery and radiotherapy
Outcomes	Overall survival and Progression-free survival (primary clinical endpoint) Adverse events (secondary clinical endpoint)	Irrelevant to time-to-event endpoints
Article types	Accessible published full text	Inaccessible full texts

^1^ RCTs: randomized controlled trials, ^2^ NSCLC: non–small cell lung cancer, M/R ^3^ HNSCC: metastatic/recurrent head and neck squamous cell carcinoma.

**Table 2:** Number of results of each keyword per hit.

Keyword	PubMed	Web of Science	Science Direct
“Afatinib” AND “Non-Small-Cell Lung Cancer”	59	782	788
“Afatinib” AND “Randomized Controlled Trial”	52	27	60
“Afatinib” AND “Squamous Cell Carcinoma of Head and Neck”	10	3	4
Total	121	812	852

**Table 3:** Grades of the quality of evidence from RCTs enrolled in meta-analysis after application of sensitivity test.

No of Studies	Study Design	Risk of Bias	Inconsistency	Indirectness	Imprecision	Publication Bias	Certainty
Overall survival in all ^1^ RCTs (assessed with: ^2^ HR)							
7	randomized trials	not serious	not serious	not serious	not serious	none	⨁⨁⨁⨁(HIGH)
Overall survival in ^3^ NSCLC RCTs (assessed with: HR)							
4	randomized trials	not serious	not serious	not serious	not serious	none	⨁⨁⨁⨁(HIGH)
Overall survival in ^4^ HNSCC RCTs (assessed with: HR)							
3	randomized trials	not serious	not serious	not serious	not serious	none	⨁⨁⨁⨁(HIGH)
Progression-free survival in all RCTs (assessed with: HR)							
6	randomized trials	not serious	not serious	not serious	not serious	none	⨁⨁⨁⨁(HIGH)
Progression-free survival in all RCTs (assessed with: HR)							
3	randomized trials	not serious	not serious	not serious	not serious	none	⨁⨁⨁⨁(HIGH)
Progression-free survival in HNSCC RCTs (assessed with: HR)							
3	randomized trials	not serious	not serious	not serious	not serious	none	⨁⨁⨁⨁(HIGH)

^1^ RCTs: randomized controlled trials, ^2^ HR: Hazard ratio, ^3^ NSCLC: non–small cell lung cancer, ^4^ HNSCC: head and neck squamous cell carcinoma.

## Data Availability

All the data are included in the manuscript and Appendix A and Appendix B.

## Appendix A

Table of study characteristics that were extracted from the included randomized controlled trials.

**Table A1:** Study characteristics extracted from the included randomized controlled trials.

ID	Study Design	Health Condition	Population	Intervention	Comparator	Outcomes		
						Overall Survival	Progression-Free Survival	Grade 3 Adverse Events (AE)
[22]	Phase III, parallel, multicenter (86 sites in 15 countries), double-blind RCT LUX-Lung 1, NCT00656136 Allocation ratio 2:1	IIIB or IV NSCLC	≥18 years, both genders, white, East Asian, other Asians and others *n* = 585	Second-line afatinib (P.O. 50 mg once daily) *n* = 390	Placebo (P.O. once daily) *n* = 195	Non-significantly different Median in intervention: 10.8 months Median in comparator 12 months	Significantly longer with afatinib Median in intervention: 3·3 months Median in comparator: 1.1 month	Dyspnea (15%)
[23]	Phase III, parallel, multicenter (133 sites in 25 countries), open-label RCT LUX-Lung 3, NCT009496500 Allocation ratio 2:1	IIIB or IV NSCLC	≥18 years, both genders, white and Eastern Asian *n* = 345	First line afatinib (P.O. 40 mg, once daily) *n* = 229	Cisplatin (i.v. 75 mg/m2) and pemetrexed (i.v. 500 mg/m^2^), once every 21 days *n* = 111	Non-significantly different, Median in intervention: 16.6months Median in comparator 14.8 months	Significantly longer in afatinib Median in intervention: 11.1 months Median in comparator 6.9 months	No severe AE were reported
[30]	Phase II, parallel, multicenter (43 sites in 4 countries), open-label RCT (Rig. No. was not accessible) Allocation ratio: 1:1	R/M HNSCC	≥18 years, both genders, Black, White, American Indian/Alaska, Natives) *n* = 121	Second-line afatinib (P.O. 50 mg, once daily) *n* = 61	Cetuximab (250 mg/m^2^ once weekly) *n* = 60	Non-significantly different Median in intervention: 35.9 weeks Median in comparator 47.1 weeks	Non-significantly different Median in intervention: 13 weeks. Median in comparator 15 weeks.	No severe AE were reported
[24]	Phase III, parallel, multicenter (36 sites in 3 countries), open-label RCT LUX Lung 6, NCT01121393 Allocation ratio: 2:1	IIIB or IV NSCLC	≥18 years, both genders, South-East Asian, South Korean, Chinese *n* = 364	First-line afatinib (P.O. 40 mg, once daily) *n* = 242	Gemcitabine (i.v. 1000 mg/m²) plus cisplatin (i.v. 75 mg/m²) in a 3-week schedule. *n* = 122	Non-significantly different Median in intervention: 22.1 months Median in comparator 22.2 months	Significantly longer in the afatinib Median in intervention: 11 months Median in comparator 5.6 months	No severe AE were reported
[32]	Phase III, parallel, multicenter (101 sites in 19 countries), open-label RCT LUX-Head and Neck 1, NCT01345682 Allocation ratio: 2:1	R/M HNSCC	≥18 years, both genders, Non-eastern Asian, Eastern Asian *n* = 483	Second-line afatinib P.O. 40 mg once daily) *n* = 322	Methotrexate (i.v. 40 mg/m² once weekly) *n* = 161	Non-significantly different Median in intervention: 6.8 months Median in comparator 6 months	Significantly longer in the afatinib Median in intervention: 6.7 months Median in comparator 2·6 months	Dyspnea (<1%), Interstitial lung disease (<1%), Pneumonia (<1%), Tracheo-oesophageal fi stula (<1%),
[25]	Phase III, parallel, multicenter (183 sites in 23 countries), open-label RCT LUX-Lung 8, NCT01523587 Allocation ratio: 1:1	IIIB or IV NSCLC	≥18 years, both genders, Non-eastern Asian, Eastern Asian *n* = 795	Second-line afatinib (P.O. 40 mg once daily) *n* = 398	Erlotinib (P.O. 150 mg once daily) *n* = 397	Significantly improved in the afatinib Median in intervention: 7.9 months Median in comparator 6.8 months	Significantly longer in the afatinib Median in intervention: 2.6 months Median in comparator 1.9 months	No severe AE were reported
[26]	Phase II, parallel, multicenter (64 sites in 13 countries), open-label RCT LUX-Lung 7, NCT01466660 Allocation ratio: 1:1	IIIB or IV NSCLC	≥18 years, both genders, Asian, Black/African American, White *n* = 319	First-line afatinib (P.O. 40 mg once daily) *n* = 160	Gefitinib (P.O. 250 mg once daily) *n* = 159	Non-significantly different Median in intervention: 27.9 months Median in comparator 25 months	Significantly longer in the afatinib Median in intervention: 11 months Median in comparator 10.9 months	Pneumonia (1%), Acute kidney injury (1%)
[31]	Phase III, parallel, multicenter (53 sites in 8 countries), open-label RCT LUX-Head & Neck 3, NCT01856478. Allocation ratio: 2:1	R/M HNSCC	≥18 years. both genders, Asian, White *n* = 340	Second-line afatinib (P.O. 40 mg once daily) *n* = 228	Methotrexate (i.v. 40 mg/m^2^, once weekly) *n* = 112	Non-significantly different Median in the intervention? months Median in comparator? months	Significantly longer in the afatinib Median in intervention: 2.9 months Median in comparator 2.6 months	No severe AE were reported

?: it was questionable because the median follow-up duration was not reported. P.O.: oral, i.v.: intravenous.

## Appendix B

Table of the extracted hazards ratios of Progression-free survival and overall survival to proceed meta-analysis.

**Table A2:** The extracted hazards ratios of Progression-free survival and overall survival to proceed meta-analysis.

	Participants			Overall Survival			Progression–Free Survival		
Study ID	Total	Afatinib	Control	Hazard Ratio	95% Confidence Interval	*p*–Value	Hazard Ratio	95% Confidence Interval	*p*–Value
[22]	585	390	195	1.08	0.86–1.35	0.74	0.38	0.31–0.48	0.0001
[23]	345	229	111	1.12	0.73–1.73	0.60	0.58	0.43–0.78	0.0010
[30]	121	61	60	1.06	0.70–1.62	0.78	0.93	0.62–1.38	0.7100
[24]	364	242	122	0.95	0.68–1.33	0.76	0.26	0.19–0.36	0.0001
[32]	483	322	161	0.96	0.77–1.19	0.70	0.80	0.65–0.98	0.0300
[25]	795	398	397	0.81	0.69–0.95	0.01	0.81	0.69–0.96	0.0103
[26]	319	160	159	0.87	0.66–1.15	0.33	0.73	0.57–0.95	0.0170
[31]	340	228	112	0.88	0.68–1.13	0.32	0.63	0.48–0.82	0.0005
